# P-1119. Air Monitoring to Detect Communicable Respiratory Viruses in Long-term Care Facilities

**DOI:** 10.1093/ofid/ofaf695.1314

**Published:** 2026-01-11

**Authors:** Tola Ewers, Shelby O’Connor, David O’Connor, Isla Emmen, Caitlyn R Kurtz, Sally Jolles, Christopher J Crnich

**Affiliations:** University of Wisconsin-Madison School of Medicine and Public Health, Madison, WI; University of Wisconsin-Madison School of Medicine and Public Health, Madison, WI; University of Wisconsin-Madison, Madison, WI; University of Wisconsin-Madison, Madison, WI; University of Wisconsin - Madison, Madison, Wisconsin; University of Wisconsin School of Medicine and Public Health, Madison, WI; University of Wisconsin School of Medicine and Public Health, Madison, WI

## Abstract

**Background:**

Respiratory viral outbreaks are a major cause of nursing home (NH) resident morbidity and mortality. These outbreaks are often seeded by infected staff who have minimal or no symptoms at the beginning of their illness. We piloted air monitoring for viral nucleic acids to evaluate whether it can predict healthcare worker infections caused by common respiratory viruses before they become clinically apparent.

**Figure 1.**
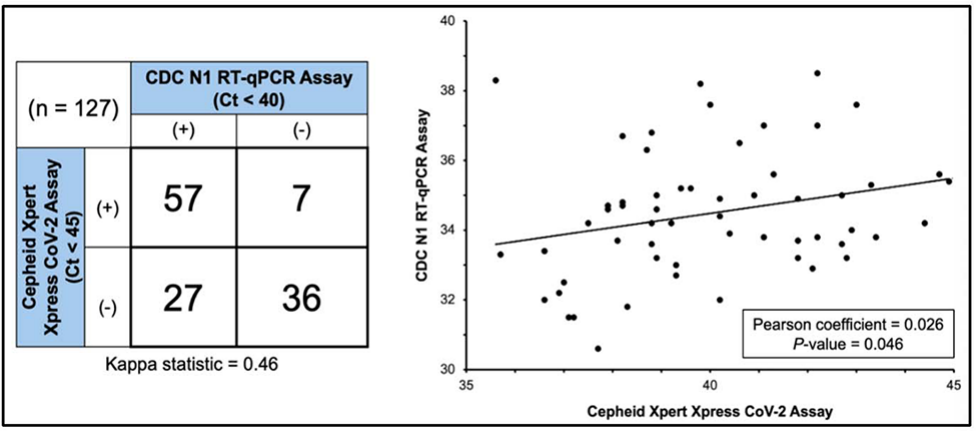
Comparison of air sample tested using on-site (Cepheid Xpert) and laboratory-based (CDC) real-time reverse transcription PCR (rRT-PCR) assays. The 2x2 table on the left demonstrates moderate correlation between both methods when looking at all results. The graph on the right demonstrates a significant correlation between the positive results obtained on both assays (n = 57).

**Figure 2.**
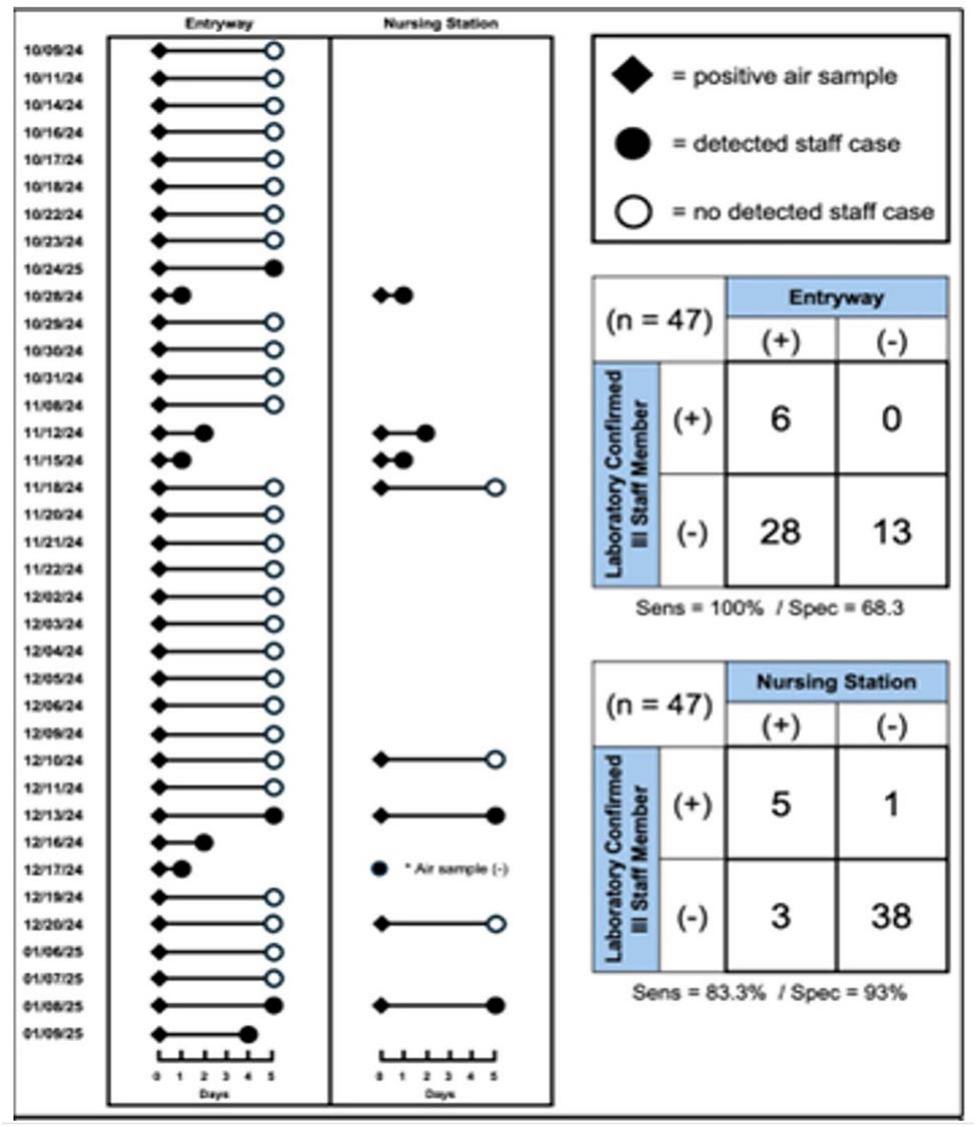
Comparison of positive air sample results collected in parallel at CLC entryway and nursing station.

Positive air samples (solid diamond), whether a laboratory-confirmed CLC staff illness was (solid circle) or was not (open circle) identified in the subsequent five days and between a positive air sample and staff result (line connecting diamonds and circles) denoted in left figure. Sensitivity and specificity for prediction of staff illnesses displayed in the 2x2 tables on the right.

**Methods:**

Air sampling units were placed in the entryway and nursing station of a single Midwest Veterans Administration Community Living Center (CLC). Air samples were collected daily (Su-Th) July 2024-Jan 2025. A commercial point-of-care reverse transcription PCR (RT-PCR) platform (Cepheid GenXpert) was used to test samples on-site, and its validity was assessed through parallel testing using a laboratory-based qRT-PCR assay. Research staff identified cases of acute respiratory illness (ARI) among unit staff and compared these data with air sample results.

**Results:**

Analyses of 127 paired air samples showed moderate levels of agreement (Kappa = 0.46) between the on-site and laboratory-based PCR assays (Figure 1). Six CLC staff developed an ARI during the study period. Entryway air samples were while highly sensitive (100.0%) for subsequent identification of a staff ARI but lacked specificity (68.3%; Figure 2). In contrast, nursing station air samples demonstrated good sensitivity (83.3%) as well as excellent specificity (93%; Figure 2). The average time interval between a positive air sample and a staff ARI was 2.8 days (range: 1–5 days).

**Conclusion:**

On-site point-of-care testing of environmental air samples reliably detected the presence of respiratory viruses in the CLC environment. Results of air samples collected in/near nursing stations appear to be more actionable than those collected at unit entryways. Environmental air samples were frequently positive several days before a staff ARI became clinically apparent suggesting this surveillance approach may help prevent or reduce the size of respiratory viral outbreaks in NHs. Further studies in different long-term care environments are needed to demonstrate the feasibility and benefits of air monitoring in NHs.

**Disclosures:**

Sally Jolles, MA, MS, Merck: Grant/Research Support Christopher J. Crnich, MD, PhD, Merck: Grant/Research Support

